# Supplementary figures and images for: Internal calibration for opportunistic computed tomography muscle density analysis
Source: PLoS One. 2022 Oct 17;17(10):e0273203. doi: 10.1371/journal.pone.0273203 (PMC9576101; doi:10.1371/journal.pone.0273203)

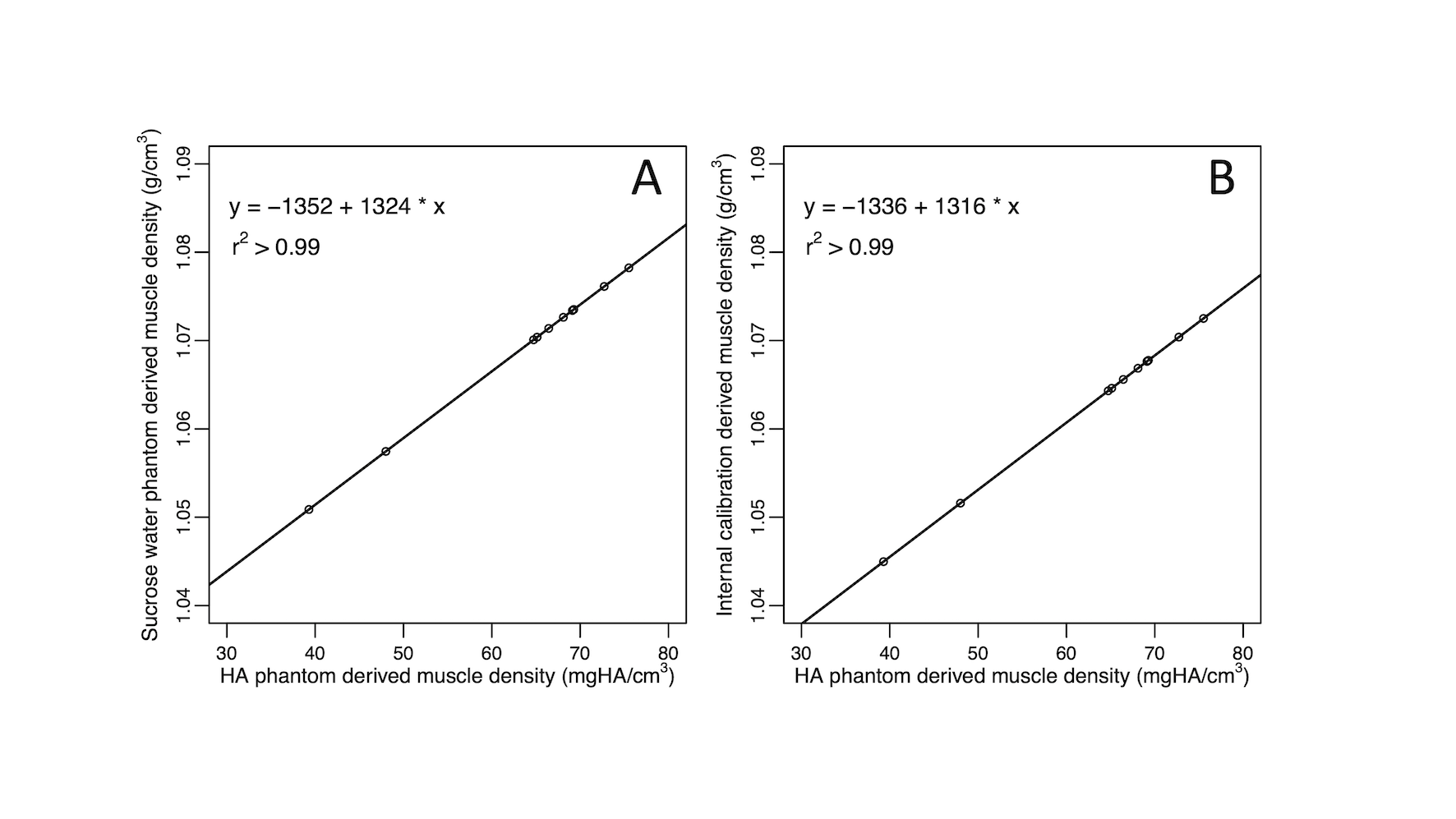

Supplement: S1 Fig — Regression plots comparing the muscle density values derived from the hydroxyapatite (HA) bone phantom method with A) the reference sucrose water phantom method and B) the internal calibration method (n = 10 muscle samples). Solid line indicates regression line, dashed line indicates the line of unity. (TIFF) [file pone.0273203.s001.tiff]

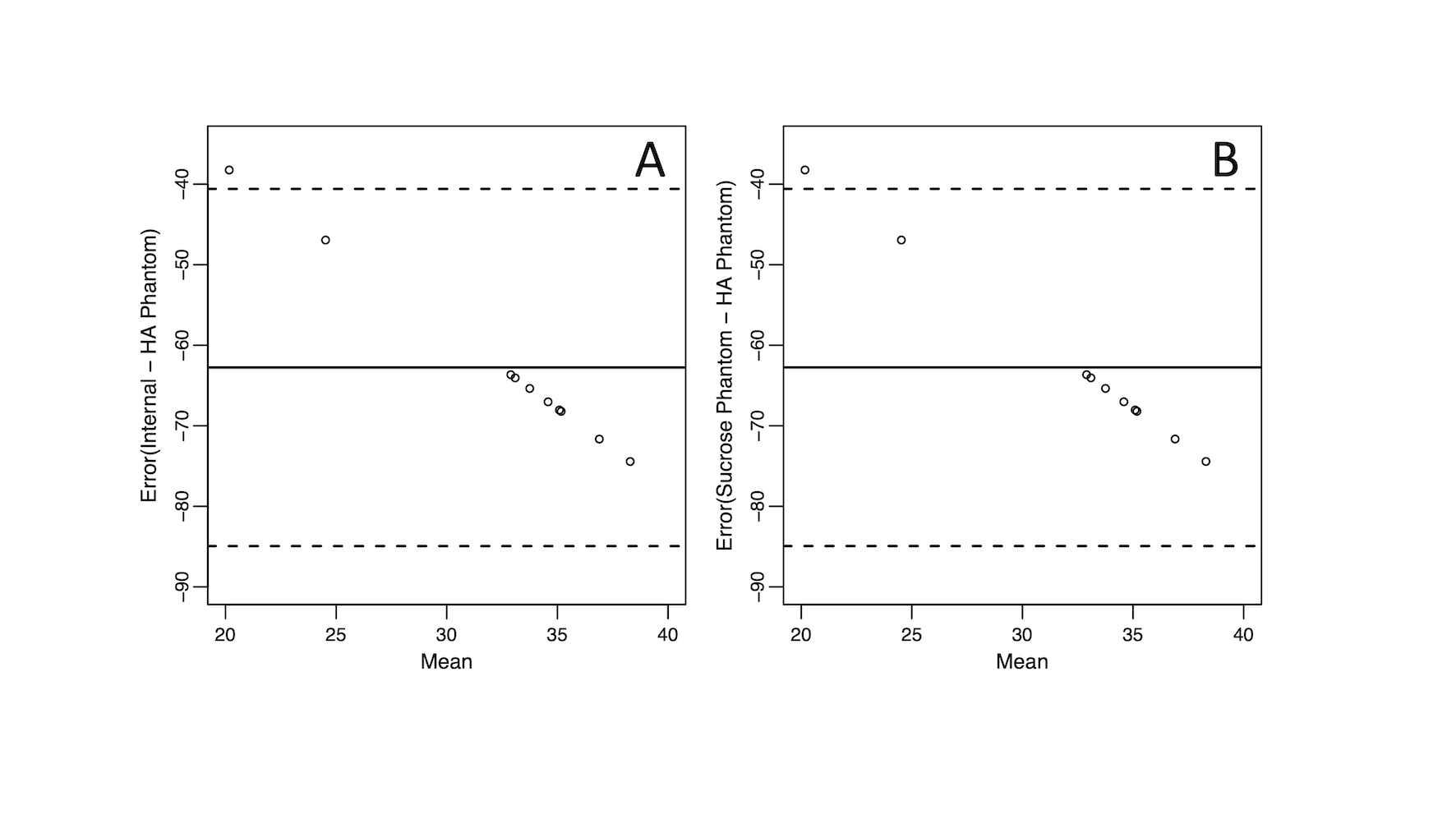

Supplement: S2 Fig — Bland-Altman plots comparing calibration methods. The mean differences are shown as solid lines and 95% limits of agreement are shown as dashed lines. A) Bland-Altman plot comparing differences between internal derived muscle density values and hydroxyapatite (HA) bone phantom derived muscle density values (n = 10 muscle samples). B) Bland-Altman plot comparing differences between the sucrose water phantom derived muscle density values and hydroxyapatite (HA) bone phantom derived muscle density values (n = 10 muscle samples). Internal and sucrose water phantom derived muscle density values are represented in g/cm3. HA phantom derived muscle density values are represented in mgHA/cm3. (TIFF) [file pone.0273203.s002.tiff]
