# Supplementary material for: Internal calibration for opportunistic computed tomography muscle density analysis
Source: PLoS One. 2022 Oct 17;17(10):e0273203. doi: 10.1371/journal.pone.0273203 (PMC9576101; doi:10.1371/journal.pone.0273203)
Supplement: S2 File — (DOCX) [file pone.0273203.s004.docx]

Internal Calibration Muscle Density Analysis

Ainsley C.J. Smith

02/08/2022

## Import Data and Libraries

mdata <- read.csv("~/Desktop//MUSPHAN/MUSPHAN_MANUSCRIPT/Data/MUSPHAN_DATA.csv", stringsAsFactors=TRUE)
View(mdata)
library(dplyr)

##
## Attaching package: 'dplyr'

## The following objects are masked from 'package:stats':
##
## filter, lag

## The following objects are masked from 'package:base':
##
## intersect, setdiff, setequal, union

library(ggplot2)
library(gridExtra)

##
## Attaching package: 'gridExtra'

## The following object is masked from 'package:dplyr':
##
## combine

library(reshape2)
library(rstatix)

##
## Attaching package: 'rstatix'

## The following object is masked from 'package:stats':
##
## filter

## Determine Coefficient of Variation Values

# Convert to numeric
mdata$Label_Id <- as.numeric(mdata$Label_Id)
mdata$Image_mean <- as.numeric(mdata$Image_mean)

#filter HU based on Label_Id
mdata_1_HU <- mdata %>% filter(Label_Id == "1", Calibration == "HU")
mdata_2_HU <- mdata %>% filter(Label_Id == "2", Calibration == "HU")
mdata_3_HU <- mdata %>% filter(Label_Id == "3", Calibration == "HU")
mdata_4_HU <- mdata %>% filter(Label_Id == "4", Calibration == "HU")
mdata_5_HU <- mdata %>% filter(Label_Id == "5", Calibration == "HU")
mdata_6_HU <- mdata %>% filter(Label_Id == "6", Calibration == "HU")
mdata_7_HU <- mdata %>% filter(Label_Id == "7", Calibration == "HU")
mdata_8_HU <- mdata %>% filter(Label_Id == "8", Calibration == "HU")
mdata_9_HU <- mdata %>% filter(Label_Id == "9", Calibration == "HU")
mdata_10_HU <- mdata %>% filter(Label_Id == "10", Calibration == "HU")

#filter Phantomless (internal) based on Label_Id
mdata_1_I <- mdata %>% filter(Label_Id == "1", Calibration == "Phantomless")
mdata_2_I <- mdata %>% filter(Label_Id == "2", Calibration == "Phantomless")
mdata_3_I <- mdata %>% filter(Label_Id == "3", Calibration == "Phantomless")
mdata_4_I <- mdata %>% filter(Label_Id == "4", Calibration == "Phantomless")
mdata_5_I <- mdata %>% filter(Label_Id == "5", Calibration == "Phantomless")
mdata_6_I <- mdata %>% filter(Label_Id == "6", Calibration == "Phantomless")
mdata_7_I <- mdata %>% filter(Label_Id == "7", Calibration == "Phantomless")
mdata_8_I <- mdata %>% filter(Label_Id == "8", Calibration == "Phantomless")
mdata_9_I <- mdata %>% filter(Label_Id == "9", Calibration == "Phantomless")
mdata_10_I <- mdata %>% filter(Label_Id == "10", Calibration == "Phantomless")

#filter AAPhantomless based on Label_Id
mdata_1_AA <- mdata %>% filter(Label_Id == "1", Calibration == "AAPhantomless")
mdata_2_AA <- mdata %>% filter(Label_Id == "2", Calibration == "AAPhantomless")
mdata_3_AA <- mdata %>% filter(Label_Id == "3", Calibration == "AAPhantomless")
mdata_4_AA <- mdata %>% filter(Label_Id == "4", Calibration == "AAPhantomless")
mdata_5_AA <- mdata %>% filter(Label_Id == "5", Calibration == "AAPhantomless")
mdata_6_AA <- mdata %>% filter(Label_Id == "6", Calibration == "AAPhantomless")
mdata_7_AA <- mdata %>% filter(Label_Id == "7", Calibration == "AAPhantomless")
mdata_8_AA <- mdata %>% filter(Label_Id == "8", Calibration == "AAPhantomless")
mdata_9_AA <- mdata %>% filter(Label_Id == "9", Calibration == "AAPhantomless")
mdata_10_AA <- mdata %>% filter(Label_Id == "10", Calibration == "AAPhantomless")

#filter Phantom based on Label_Id
mdata_1_P <- mdata %>% filter(Label_Id == "1", Calibration == "Phantom")
mdata_2_P <- mdata %>% filter(Label_Id == "2", Calibration == "Phantom")
mdata_3_P <- mdata %>% filter(Label_Id == "3", Calibration == "Phantom")
mdata_4_P <- mdata %>% filter(Label_Id == "4", Calibration == "Phantom")
mdata_5_P <- mdata %>% filter(Label_Id == "5", Calibration == "Phantom")
mdata_6_P <- mdata %>% filter(Label_Id == "6", Calibration == "Phantom")
mdata_7_P <- mdata %>% filter(Label_Id == "7", Calibration == "Phantom")
mdata_8_P <- mdata %>% filter(Label_Id == "8", Calibration == "Phantom")
mdata_9_P <- mdata %>% filter(Label_Id == "9", Calibration == "Phantom")
mdata_10_P <- mdata %>% filter(Label_Id == "10", Calibration == "Phantom")

# calculate the coefficient of variation across scan protocols and positions
cv_1_HU <- sd(mdata_1_HU$Image_mean) / mean(mdata_1_HU$Image_mean) * 100
cv_2_HU <- sd(mdata_2_HU$Image_mean) / mean(mdata_2_HU$Image_mean) * 100
cv_3_HU <- sd(mdata_3_HU$Image_mean) / mean(mdata_3_HU$Image_mean) * 100
cv_4_HU <- sd(mdata_4_HU$Image_mean) / mean(mdata_4_HU$Image_mean) * 100
cv_5_HU <- sd(mdata_5_HU$Image_mean) / mean(mdata_5_HU$Image_mean) * 100
cv_6_HU <- sd(mdata_6_HU$Image_mean) / mean(mdata_6_HU$Image_mean) * 100
cv_7_HU <- sd(mdata_7_HU$Image_mean) / mean(mdata_7_HU$Image_mean) * 100
cv_8_HU <- sd(mdata_8_HU$Image_mean) / mean(mdata_8_HU$Image_mean) * 100
cv_9_HU <- sd(mdata_9_HU$Image_mean) / mean(mdata_9_HU$Image_mean) * 100
cv_10_HU <- sd(mdata_10_HU$Image_mean) / mean(mdata_10_HU$Image_mean) * 100

cv_1_I <- sd(mdata_1_I$Image_mean) / mean(mdata_1_I$Image_mean) * 100
cv_2_I <- sd(mdata_2_I$Image_mean) / mean(mdata_2_I$Image_mean) * 100
cv_3_I <- sd(mdata_3_I$Image_mean) / mean(mdata_3_I$Image_mean) * 100
cv_4_I <- sd(mdata_4_I$Image_mean) / mean(mdata_4_I$Image_mean) * 100
cv_5_I <- sd(mdata_5_I$Image_mean) / mean(mdata_5_I$Image_mean) * 100
cv_6_I <- sd(mdata_6_I$Image_mean) / mean(mdata_6_I$Image_mean) * 100
cv_7_I <- sd(mdata_7_I$Image_mean) / mean(mdata_7_I$Image_mean) * 100
cv_8_I <- sd(mdata_8_I$Image_mean) / mean(mdata_8_I$Image_mean) * 100
cv_9_I <- sd(mdata_9_I$Image_mean) / mean(mdata_9_I$Image_mean) * 100
cv_10_I <- sd(mdata_10_I$Image_mean) / mean(mdata_10_I$Image_mean) * 100

cv_1_AA <- sd(mdata_1_AA$Image_mean) / mean(mdata_1_AA$Image_mean) * 100
cv_2_AA <- sd(mdata_2_AA$Image_mean) / mean(mdata_2_AA$Image_mean) * 100
cv_3_AA <- sd(mdata_3_AA$Image_mean) / mean(mdata_3_AA$Image_mean) * 100
cv_4_AA <- sd(mdata_4_AA$Image_mean) / mean(mdata_4_AA$Image_mean) * 100
cv_5_AA <- sd(mdata_5_AA$Image_mean) / mean(mdata_5_AA$Image_mean) * 100
cv_6_AA <- sd(mdata_6_AA$Image_mean) / mean(mdata_6_AA$Image_mean) * 100
cv_7_AA <- sd(mdata_7_AA$Image_mean) / mean(mdata_7_AA$Image_mean) * 100
cv_8_AA <- sd(mdata_8_AA$Image_mean) / mean(mdata_8_AA$Image_mean) * 100
cv_9_AA <- sd(mdata_9_AA$Image_mean) / mean(mdata_9_AA$Image_mean) * 100
cv_10_AA <- sd(mdata_10_AA$Image_mean) / mean(mdata_10_AA$Image_mean) * 100

cv_1_P <- sd(mdata_1_P$Image_mean) / mean(mdata_1_P$Image_mean) * 100
cv_2_P <- sd(mdata_2_P$Image_mean) / mean(mdata_2_P$Image_mean) * 100
cv_3_P <- sd(mdata_3_P$Image_mean) / mean(mdata_3_P$Image_mean) * 100
cv_4_P <- sd(mdata_4_P$Image_mean) / mean(mdata_4_P$Image_mean) * 100
cv_5_P <- sd(mdata_5_P$Image_mean) / mean(mdata_5_P$Image_mean) * 100
cv_6_P <- sd(mdata_6_P$Image_mean) / mean(mdata_6_P$Image_mean) * 100
cv_7_P <- sd(mdata_7_P$Image_mean) / mean(mdata_7_P$Image_mean) * 100
cv_8_P <- sd(mdata_8_P$Image_mean) / mean(mdata_8_P$Image_mean) * 100
cv_9_P <- sd(mdata_9_P$Image_mean) / mean(mdata_9_P$Image_mean) * 100
cv_10_P <- sd(mdata_10_P$Image_mean) / mean(mdata_10_P$Image_mean) * 100

# calculate the mean coefficient of variation for each calibration method
na.rm=TRUE
mean_CV_HU <- mean(cv_1_HU, cv_2_HU, cv_3_HU, cv_4_HU, cv_5_HU, cv_6_HU, cv_7_HU, cv_8_HU, cv_9_HU, cv_10_HU)
mean_CV_I <- mean(cv_1_I, cv_2_I, cv_3_I, cv_4_I, cv_5_I, cv_6_I, cv_7_I, cv_8_I, cv_9_I, cv_10_I)
mean_CV_AA <- mean(cv_1_AA, cv_2_AA, cv_3_AA, cv_4_AA, cv_5_AA, cv_6_AA, cv_7_AA, cv_8_AA, cv_9_AA, cv_10_AA)
mean_CV_P <- mean(cv_1_P, cv_2_P, cv_3_P, cv_4_P, cv_5_P, cv_6_P, cv_7_P, cv_8_P, cv_9_P, cv_10_P)

#total muscle density mean and SD for each calibration method; scan 4 (centre position, chest)
df2 <- mdata[!(mdata$Label_Id==0),]
mdata <- df2 %>% filter(Musphan == "MUSPHAN_004")
mdataHU <-mdata %>% filter(Calibration == "HU")
mean(mdataHU$Image_mean)

## [1] 57.10773

sd(mdataHU$Image_mean)

## [1] 8.55072

mdataAA <-mdata %>% filter(Calibration == "AAPhantomless")
mean(mdataAA$Image_mean)

## [1] 1.063622

sd(mdataAA$Image_mean)

## [1] 0.008604812

mdataP <-mdata %>% filter(Calibration == "Phantom")
mean(mdataP$Image_mean)

## [1] 1.069408

sd(mdataP$Image_mean)

## [1] 0.00855072

## Anova, Bonferroni, and Boxplots for Coefficient of Variation

##import CV data (same as was generated above)
cvdata <- read.csv("~/Desktop//MUSPHAN/MUSPHAN_MANUSCRIPT/Data/MUSPHAN_CV.csv", stringsAsFactors=TRUE)
View(cvdata)
dataSetName <- "CV Calibration Muscle Density Analysis"

#within subject anova, measure sphericity
Calibration=factor(cvdata$Calibration)
CV=cvdata$CV
Label_Id=cvdata$Label_Id
anova1 <- anova_test(data = cvdata, dv = CV, wid = Label_Id, within = Calibration)
anova1

## ANOVA Table (type III tests)
##
## $ANOVA
## Effect DFn DFd F p p<.05 ges
## 1 Calibration 2 18 76.532 1.58e-09 * 0.842
##
## $`Mauchly's Test for Sphericity`
## Effect W p p<.05
## 1 Calibration 0.001 2.45e-12 *
##
## $`Sphericity Corrections`
## Effect GGe DF[GG] p[GG] p[GG]<.05 HFe DF[HF] p[HF] p[HF]<.05
## 1 Calibration 0.5 1, 9.01 1.07e-05 * 0.5 1, 9.01 1.07e-05 *

#bonferroni post-hoc
pairwise.t.test(CV, Calibration, p.adj='bonferroni')

##
## Pairwise comparisons using t tests with pooled SD
##
## data: CV and Calibration
##
## HU Internal
## Internal 1.4e-10 -
## Phantom 2.8e-10 1
##
## P value adjustment method: bonferroni

#boxplot setup
file = paste(dataSetName,".pdf",sep="")
pdf(file, width = 8.5, height = 8.5)
par(mfrow=c(2,2),oma=c(0,4,4,6),pty='s')
data <- melt(cvdata, id.vars = c("Label_Id", "Calibration"), variable.name = "CV",
 value.name = "Calibration")

#making the boxplot
ggplot(cvdata, aes(y=CV,x=reorder(Calibration, -CV),color=Calibration, fill=Calibration))+
 geom_boxplot(alpha=0.5, outlier.alpha = 0) +
 scale_fill_manual(values=c('blue','green', 'yellow'),aesthetics="fill")+
 scale_fill_manual(values=c('blue4','green4', 'yellow4'),aesthetics="color")+
 geom_jitter(aes(color=factor(Calibration)))+
 theme(panel.grid.major = element_blank(),panel.grid.minor = element_blank(),panel.background = element_rect(fill="white"), legend.position="none", axis.text=element_text(size=20,colour = "black"), axis.title=element_text(size=20), panel.border = element_rect(fill=NA, size =1.5))+
 ylab("CV (%)") + xlab("")
dev.off()

## quartz_off_screen
## 2

## Regression and Bland-Altmans Internal V Phantom

source("~/Desktop//MUSPHAN/MUSPHAN_MANUSCRIPT/Analysis/RegressionNoEqn.R")
source("~/Desktop//MUSPHAN/MUSPHAN_MANUSCRIPT/Analysis/BlandAltmanHorizontalNoLabel.R")
source("~/Desktop//MUSPHAN/MUSPHAN_MANUSCRIPT/Analysis/BlandAltmanHorizontalMultiColour.R")

dataSetName <- "Internal Calibration ROIs Muscle Density Analysis"

# Only scan 4, no Label_Id=0
df2 <- mdata[!(mdata$Label_Id==0),]
mdata4 <- df2 %>% filter(Musphan == "MUSPHAN_004")
View(mdata4)

# Plot Settings
szVarName = 1
szLetter = 1.5
sz=0.9; #axis labels
szout = 1.1;
sz1=0.8; #axis numbers r
sz2=0.8 #regression labels
sz3=0.2; #Bland-Altman labels
szPoint = 0.7 #size of data point

file = paste(dataSetName,".pdf",sep="")
pdf(file, width = 8.5, height = 8.5)
par(mfrow=c(2,2),oma=c(0,4,4,6),pty='s')

#Prepare data
varName = mdata4$Image_mean

mdatavarName1 = mdata4 %>% filter(Calibration == "Phantom")
varName1 = mdatavarName1$Image_mean

mdatavarName2 = mdata4 %>% filter(Calibration == "AAPhantomless")
varName2 = mdatavarName2$Image_mean

mdatavarName3 = mdata4 %>% filter(Calibration == "Phantomless")
varName3 = mdatavarName3$Image_mean

mdatavarName4 = mdata4 %>% filter(Calibration == "NBPhantomless")
varName4 = mdatavarName4$Image_mean

mdatavarName5 = mdata4 %>% filter(Calibration == "ABPhantomless")
varName5 = mdatavarName5$Image_mean

mdatavarName6 = mdata4 %>% filter(Calibration == "AABPhantomless")
varName6 = mdatavarName6$Image_mean

mdatavarName7 = mdata4 %>% filter(Calibration == "HAPhantom")
varName7 = mdatavarName7$Image_mean

## REGRESSION SUCROSE PHANTOM V INTERNAL AA
 lowlim1 = 1.04; #lowest value on y-axis
 lowlim2 = 1.04; #highest value on x-axis
 highlim1 = 1.09; #highest value on y-axis
 highlim2 = 1.09; #highest value on x-axis
 ylim1=0.010;offset=0; #ylim is the maximum expected error on the Bland-Altman plot
 Regression(varName1,varName2,expression(paste("Phantom derived muscle density (g/cm"^"3",")")),expression(paste("Internal derived muscle density (g/cm"^"3",")")),"",0)
 #Add R^2 value to plot
 output = lm(varName1 ~ varName2)
 summary(output)

##
## Call:
## lm(formula = varName1 ~ varName2)
##
## Residuals:
## Min 1Q Median 3Q Max
## -7.783e-06 -9.974e-07 1.182e-06 2.886e-06 3.726e-06
##
## Coefficients:
## Estimate Std. Error t value Pr(>|t|)
## (Intercept) 0.0124721 0.0001759 70.89 1.75e-12 ***
## varName2 0.9937136 0.0001654 6007.56 < 2e-16 ***
## ---
## Signif. codes: 0 '***' 0.001 '**' 0.01 '*' 0.05 '.' 0.1 ' ' 1
##
## Residual standard error: 4.27e-06 on 8 degrees of freedom
## Multiple R-squared: 1, Adjusted R-squared: 1
## F-statistic: 3.609e+07 on 1 and 8 DF, p-value: < 2.2e-16

text(1.045, 1.081, expression(paste("r"^"2"," > 0.99")), cex=szVarName)
 text(1.051, 1.085, expression(paste("y = 0.01 + 0.99 * x")))

## BLAND ALTMANS

#SUCROSE PHANTOM VS All ROI COMBINATIONS
 lowlim1 = 1.02; #lowest value on x-axis
 highlim1 = 1.08; #highest value on x-axis
 ylim1=(0.06);offset=0; #ylim is the maximum expected error on the Bland-Altman plot
 ylim2=(0.02)

 xlab2= "(Mean (g/cm^3~))"
 ylab2= "Error(Internal-Phantom) (g/cm^3~)"
 plot(varName1,varName2,type = "n",ylim=c(-ylim1, ylim2),xlim=c(lowlim1,highlim1),xlab=expression("Mean (g/cm"^"3"*")"),ylab=expression("Error(Internal-Phantom) (g/cm"^"3"*")"),
 cex.lab=sz, cex.axis=sz1, cex.sub=sz,cex=szPoint,mgp=c(1.5,0.5,0))
 BlandAltmanColour(varName1,varName2,"red",varName)

##
## Call:
## lm(formula = diff ~ ave)
##
## Coefficients:
## (Intercept) ave
## -0.012511 0.006306
##
## [1] "Mean Difference -0.00578572999999996"
## [1] "95%LOA 0.000106316668554951"

BlandAltmanColour(varName1,varName3,5,varName)

##
## Call:
## lm(formula = diff ~ ave)
##
## Coefficients:
## (Intercept) ave
## 0.07844 -0.12821
##
## [1] "Mean Difference -0.05512873"
## [1] "95%LOA 0.00201925676586524"

BlandAltmanColour(varName1,varName4,"blue",varName)

##
## Call:
## lm(formula = diff ~ ave)
##
## Coefficients:
## (Intercept) ave
## -0.012934 0.007946
##
## [1] "Mean Difference -0.00445472999999994"
## [1] "95%LOA 0.000133915040344247"

BlandAltmanColour(varName1,varName5,"purple",varName)

##
## Call:
## lm(formula = diff ~ ave)
##
## Coefficients:
## (Intercept) ave
## -0.012204 0.007318
##
## [1] "Mean Difference -0.00439472999999995"
## [1] "95%LOA 0.000123321786789959"

BlandAltmanColour(varName1,varName6,7,varName)

##
## Call:
## lm(formula = diff ~ ave)
##
## Coefficients:
## (Intercept) ave
## -0.012411 0.006937
##
## [1] "Mean Difference -0.00500972999999998"
## [1] "95%LOA 0.000116927541415737"

legend(1.02, -0.02, legend=c("Adipose, Air","Blood, Air", "Adipose, Air, Blood", "Adipose, Air, Blood, Muscle", "Adipose, Air, Blood, Muscle, Bone"), col=c("red","purple",7,"blue",5), lty=1:1, cex=0.7)

 #SUCROSE PHANTOM VS AB and AA ROI COMBINATIONS
 lowlim1 = 1.02; #lowest value on x-axis
 highlim1 = 1.08; #highest value on x-axis
 ylim1=(0.006);offset=0; #ylim is the maximum expected error on the Bland-Altman plot
 ylim2=(0.00001)

 xlab2= "(Mean (g/cm^3~))"
 ylab2= "Error(Internal-Phantom) (g/cm^3~)"
 plot(varName1,varName5,type = "n",ylim=c(-ylim1, ylim2),xlim=c(lowlim1,highlim1),xlab=expression("Mean (g/cm"^"3"*")"),ylab=expression("Error(Internal-Phantom) (g/cm"^"3"*")"),
 cex.lab=sz, cex.axis=sz1, cex.sub=sz,cex=szPoint,mgp=c(1.5,0.5,0))
 BlandAltmanColour(varName1,varName2,"red",varName)

##
## Call:
## lm(formula = diff ~ ave)
##
## Coefficients:
## (Intercept) ave
## -0.012511 0.006306
##
## [1] "Mean Difference -0.00578572999999996"
## [1] "95%LOA 0.000106316668554951"

BlandAltmanColour(varName1,varName5,"purple",varName)

##
## Call:
## lm(formula = diff ~ ave)
##
## Coefficients:
## (Intercept) ave
## -0.012204 0.007318
##
## [1] "Mean Difference -0.00439472999999995"
## [1] "95%LOA 0.000123321786789959"

legend(1.02, -0.001, legend=c("Adipose, Air","Blood, Air"), col=c("red","purple"), lty=1:1, cex=0.7)

 #find relative accuracy error for MUSPHAN_004
 View(mdata4)
 meanPhantom = mean(varName1)
 meanAAPhantomless = mean(varName2)
 relative_accuracy_error = (meanPhantom - meanAAPhantomless)/(meanPhantom)*100

 ###########################################

dataSetName <- "HA Phantom Calibration"

# Plot Settings
szVarName = 1
szLetter = 1.5
sz=0.9; #axis labels
szout = 1.1;
sz1=0.8; #axis numbers r
sz2=0.8 #regression labels
sz3=0.2; #Bland-Altman labels
szPoint = 0.7 #size of data point

file = paste(dataSetName,".pdf",sep="")
pdf(file, width = 8.5, height = 8.5)
par(mfrow=c(2,2),oma=c(0,4,4,6),pty='s')

 ## REGRESSION HA PHANTOM V SUCROSE PHANTOM
 lowlim1 = 1.04; #lowest value on y-axis
 lowlim2 = 30; #highest value on x-axis
 highlim1 = 1.09; #highest value on y-axis
 highlim2 = 80; #highest value on x-axis
 ylim1=0.010;offset=0; #ylim is the maximum expected error on the Bland-Altman plot
 Regression(varName7,varName1,expression(paste("HA phantom derived muscle density (mgHA/cm"^"3",")")),expression(paste("Sucrose water phantom derived muscle density (g/cm"^"3",")")),"",0)

## Warning in summary.lm(compare.lm): essentially perfect fit: summary may be
## unreliable

## Warning in summary.lm(compare.lm): essentially perfect fit: summary may be
## unreliable

## Warning in summary.lm(compare.lm): essentially perfect fit: summary may be
## unreliable

## Warning in summary.lm(compare.lm): essentially perfect fit: summary may be
## unreliable

#Add R^2 value to plot
 output = lm(varName7 ~ varName1)
 summary(output)

##
## Call:
## lm(formula = varName7 ~ varName1)
##
## Residuals:
## Min 1Q Median 3Q Max
## -5.316e-13 -5.240e-15 5.853e-14 1.235e-13 1.413e-13
##
## Coefficients:
## Estimate Std. Error t value Pr(>|t|)
## (Intercept) -1.352e+03 8.880e-12 -1.523e+14 <2e-16 ***
## varName1 1.324e+03 8.304e-12 1.594e+14 <2e-16 ***
## ---
## Signif. codes: 0 '***' 0.001 '**' 0.01 '*' 0.05 '.' 0.1 ' ' 1
##
## Residual standard error: 2.13e-13 on 8 degrees of freedom
## Multiple R-squared: 1, Adjusted R-squared: 1
## F-statistic: 2.542e+28 on 1 and 8 DF, p-value: < 2.2e-16

text(35.6, 1.081, expression(paste("r"^"2"," > 0.99")), cex=szVarName)
 text(42.5, 1.085, expression(paste("y = -1352 + 1324 * x")))

 ## REGRESSION HA PHANTOM V AA INTERNAL
 lowlim1 = 1.04; #lowest value on y-axis
 lowlim2 = 30; #highest value on x-axis
 highlim1 = 1.09; #highest value on y-axis
 highlim2 = 80; #highest value on x-axis
 ylim1=0.010;offset=0; #ylim is the maximum expected error on the Bland-Altman plot
 Regression(varName7,varName2,expression(paste("HA phantom derived muscle density (mgHA/cm"^"3",")")),expression(paste("Internal calibration derived muscle density (g/cm"^"3",")")),"",0)
 #Add R^2 value to plot
 output = lm(varName7 ~ varName2)
 summary(output)

##
## Call:
## lm(formula = varName7 ~ varName2)
##
## Residuals:
## Min 1Q Median 3Q Max
## -0.010305 -0.001321 0.001565 0.003821 0.004933
##
## Coefficients:
## Estimate Std. Error t value Pr(>|t|)
## (Intercept) -1335.5531 0.2329 -5733 <2e-16 ***
## varName2 1315.6768 0.2190 6008 <2e-16 ***
## ---
## Signif. codes: 0 '***' 0.001 '**' 0.01 '*' 0.05 '.' 0.1 ' ' 1
##
## Residual standard error: 0.005653 on 8 degrees of freedom
## Multiple R-squared: 1, Adjusted R-squared: 1
## F-statistic: 3.609e+07 on 1 and 8 DF, p-value: < 2.2e-16

text(35.5, 1.081, expression(paste("r"^"2"," > 0.99")), cex=szVarName)
 text(42.5, 1.085, expression(paste("y = -1336 + 1316 * x")))

## BLAND ALTMANS

#HA PHANTOM VS INTERNAL AA
 lowlim1 = 20; #lowest value on x-axis
 highlim1 = 40; #highest value on x-axis
 ylim1=(90);offset=0; #ylim is the maximum expected error on the Bland-Altman plot
 ylim2=(-35)
 xlab2= "Mean"
 ylab2= "Error(Internal - Phantom)"
 plot(varName7,varName2,type = "n",ylim=c(-ylim1, ylim2),xlim=c(lowlim1,highlim1),xlab=expression("Mean"),ylab=expression("Error(Internal - HA Phantom)"),
 cex.lab=sz, cex.axis=sz1, cex.sub=sz,cex=szPoint,mgp=c(1.5,0.5,0))
 BlandAltmanColour(varName7,varName2,"black",varName)

##
## Call:
## lm(formula = diff ~ ave)
##
## Coefficients:
## (Intercept) ave
## 2.029 -1.997
##
## [1] "Mean Difference -62.76601252"
## [1] "95%LOA 22.1725942069607"

#HA PHANTOM VS SUCROSE PHANTOM
 lowlim1 = 20; #lowest value on x-axis
 highlim1 = 40; #highest value on x-axis
 ylim1=(90);offset=0; #ylim is the maximum expected error on the Bland-Altman plot
 ylim2=(-35)
 xlab2= "Mean"
 ylab2= "Error(Internal - Phantom)"
 plot(varName7,varName1,type = "n",ylim=c(-ylim1, ylim2),xlim=c(lowlim1,highlim1),xlab=expression("Mean"),ylab=expression("Error(Sucrose Phantom - HA Phantom)"),
 cex.lab=sz, cex.axis=sz1, cex.sub=sz,cex=szPoint,mgp=c(1.5,0.5,0))
 BlandAltmanColour(varName7,varName1,"black",varName)

## Warning in summary.lm(lm_BA): essentially perfect fit: summary may be unreliable

##
## Call:
## lm(formula = diff ~ ave)
##
## Coefficients:
## (Intercept) ave
## 2.041 -1.997
##
## [1] "Mean Difference -62.76022679"
## [1] "95%LOA 22.1727002266913"

## Warning in summary.lm(lm_BA): essentially perfect fit: summary may be unreliable

dev.off()

## quartz_off_screen
## 2

## Data Dictionary

# Position = The position of the muscle samples within the CT scanner bore (centre, right, left, top, bottom).

# Musphan = The name assigned to the CT image. A total of 10 CT images were acquired.

# Protocol = The scan protocol used to acquire the CT image.

# Chest = Chest mediastinum scan protocol.

# Abdomen = Abdominal kidney, urine, bladder (KUB) thins scan protocol.

# Calibration = The calibration method used to derive muscle density values.

# HU = Native Hounsfield Unit values. No additional calibration method applied. The corresponding “Image_mean” values are in Hounsfield Units.

# Phantomless = Internal calibration with air, adipose, blood, muscle, and bone regions of interest selected. The corresponding “Image_mean” values are in g/cm^3.

# NBPhantomless = Internal calibration with only air, adipose, blood, and muscle regions of interest selected. The corresponding “Image_mean” values are in g/cm^3.

# AAPhantomless = Internal calibration with only air and adipose regions of interest selected. The corresponding “Image_mean” values are in g/cm^3.

# Phantom = Phantom calibration with the reference sucrose water phantom. The corresponding “Image_mean” values are in g/cm^3.

# AABPhantomless = Internal calibration with only air, adipose, and blood regions of interest selected. The corresponding “Image_mean” values are in g/cm^3.

# ABPhantomless = Internal calibration with only air and blood regions of interest selected. The corresponding “Image_mean” values are in g/cm^3.

# HAPhantom = Phantom calibration with the hydroxyapatite bone phantom. The corresponding “Image_mean” values are in mg of hydroxyapatite/cm^3.

# Label_Id = The number assigned to the muscle sample. A total of 10 muscle samples (Label_Id 1-10) were included in the scans. If the Label_Id = 0, then this corresponds to the scan 'background' and not a muscle sample.

# Image_mean = The mean muscle density measured in HU, g/cm^3, or mg of hydroxyapatite/cm^3.
