# Supplementary material for: Internal calibration for opportunistic computed tomography muscle density analysis
Source: PLoS One. 2022 Oct 17;17(10):e0273203. doi: 10.1371/journal.pone.0273203 (PMC9576101; doi:10.1371/journal.pone.0273203)
Supplement: S4 File — (ZIP) [file pone.0273203.s006.zip › S6/OGO-READ-ME_muscle analysis.docx]

**Document Overview**

This document is meant to provide an overview of using the OGO Python scripts for performing opportunistic CT screening. The opportunistic screening pipeline includes image segmentation, internal density calibration, bone mineral density (BMD) analysis, finite element (FE) model generation, FE model post-processing, and results tabulation. Each of these steps will be discussed below and how to implement the analyses. This bone analysis pipeline is set up to analyze abdominal CT scans, which includes the right proximal femur, left proximal femur, right pelvis, left pelvis, sacrum, L5 vertebra, L4 vertebra, L3 vertebra, L2 vertebra, and L1 vertebra. All CT scan images can be performed using DICOM or NIFTI file formats. For purposes here, we use the NIFTI file format, as it is a single image file, rather than a directory of multiple files. All example analyses use images from the OGO_Example_Data directory included with the analysis scripts.

Included in the OGO Python scripts are some additional scripts for performing QCT phantom-based density calibration, where different scripts are for different calibration phantoms. These details are at the end of this document.

**Relevant File Naming in Example Images**

*OGO_Abdomen_Example.nii* – This is an example abdominal CT scan, converted to NIFTI format from a DICOM image sequence.

*OGO_Abdomen_Example_PERI_CORR.nii* – This is the segmentation MASK image, where each different label is a specific bone and a value of 0 is the background.

*OGO_Abdomen_Example_IC_MASK.nii* – This is the internal density calibration MASK image, where each different label is a specific region-of-interest for the calibration and a value of 0 is the background.

**Step 1: Image segmentation to identify muscle of interest**

Image segmentation can be performed using a number of different tools. For purposes here, we use manual image segmentation in ITK-SNAP. The main necessary component is to stay consistent with the proper bone labels (see included labels text file: ‘Inernal-Calibration_ITKSNAP_Labels.txt’). These bone labels are used for all analyses when applying the MASK image.

| **Label Value** | **Description** |
| --- | --- |
| 0 | Clear Label |
| 1 | Adipose |
| 2 | Air |
| 3 | Blood |
| 4 | Full Cortical Bone |
| 5 | Skeletal Muscle |

**Step 2: Internal density calibration**

The internal density calibration step uses the OGO script *ogo_internal_calibration.py*. This script reads in the base image and the internal density calibration MASK image. The internal density calibration MASK image (*OGO_Abdomen_Example_IC_MASK.nii*) is currently generated in ITK-SNAP by manual segmentation, using the provided label files (*Internal-Calibration_ITKSNAP_Labels.txt*).

| **Label Value** | **Description** |
| --- | --- |
| 0 | Clear label |
| 1 | Adipose |
| 2 | Air |
| 3 | Blood |
| 4 | Cortical Bone |
| 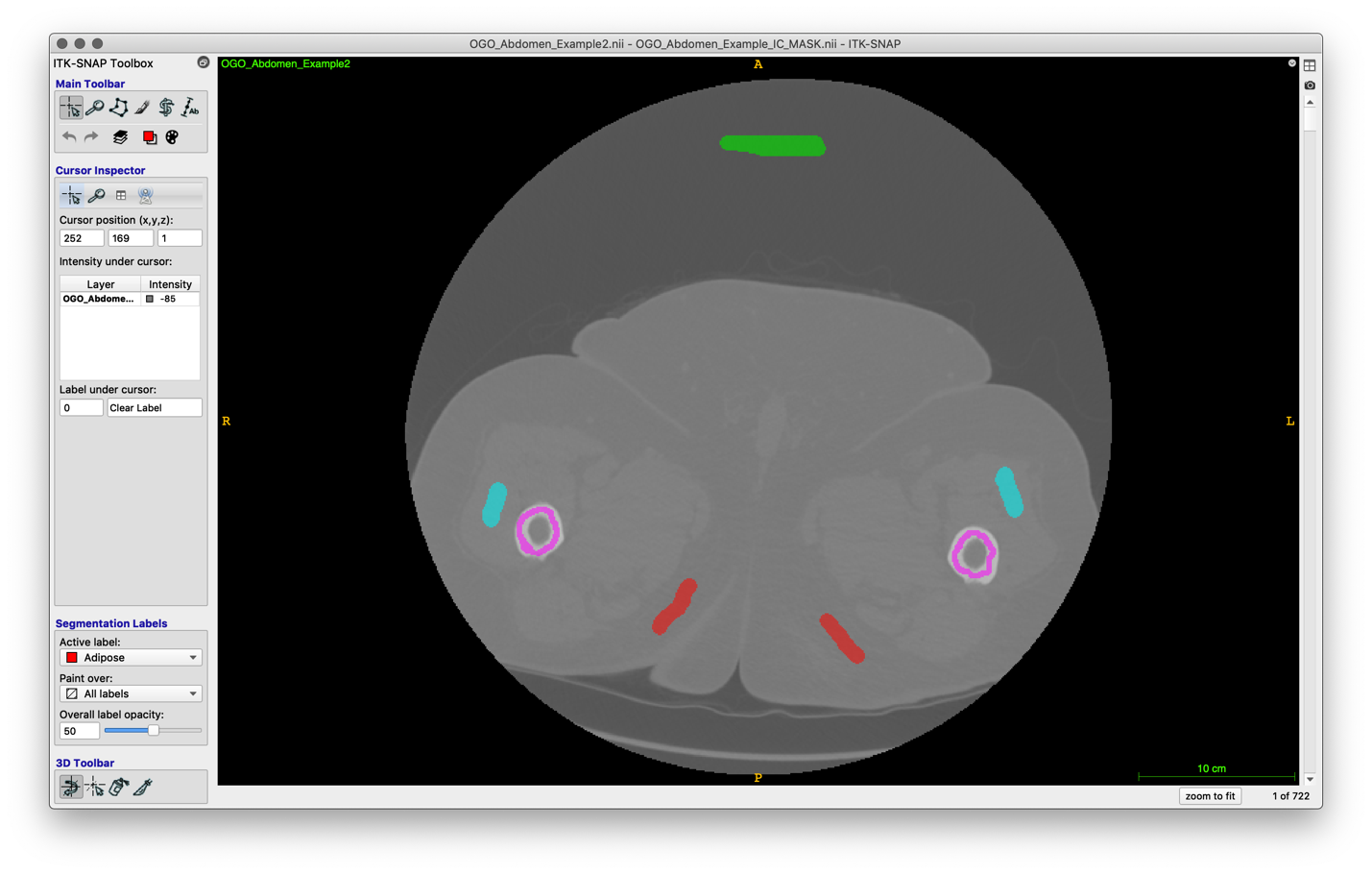5 | Skeletal Muscle |

Above is an example screenshot of the region definitions using ITK-SNAP. From ITK-SNAP, save the calibration mask as **_IC_MASK.nii* (i.e., *OGO_Abdomen_Example_IC_MASK.nii*).

The internal calibration script is run from a terminal. Activate your analysis environment and change to the OGO script directory. To run the internal calibration, use the command prompt as follows:

$ python ogo_internal_calibration PATH/TO/OGO_Example_Data/OGO_Abdomen_Example.nii PATH/TO/OGO_Example_Data/OGO_Abdomen_Example_IC_MASK.nii

OUTPUTS:


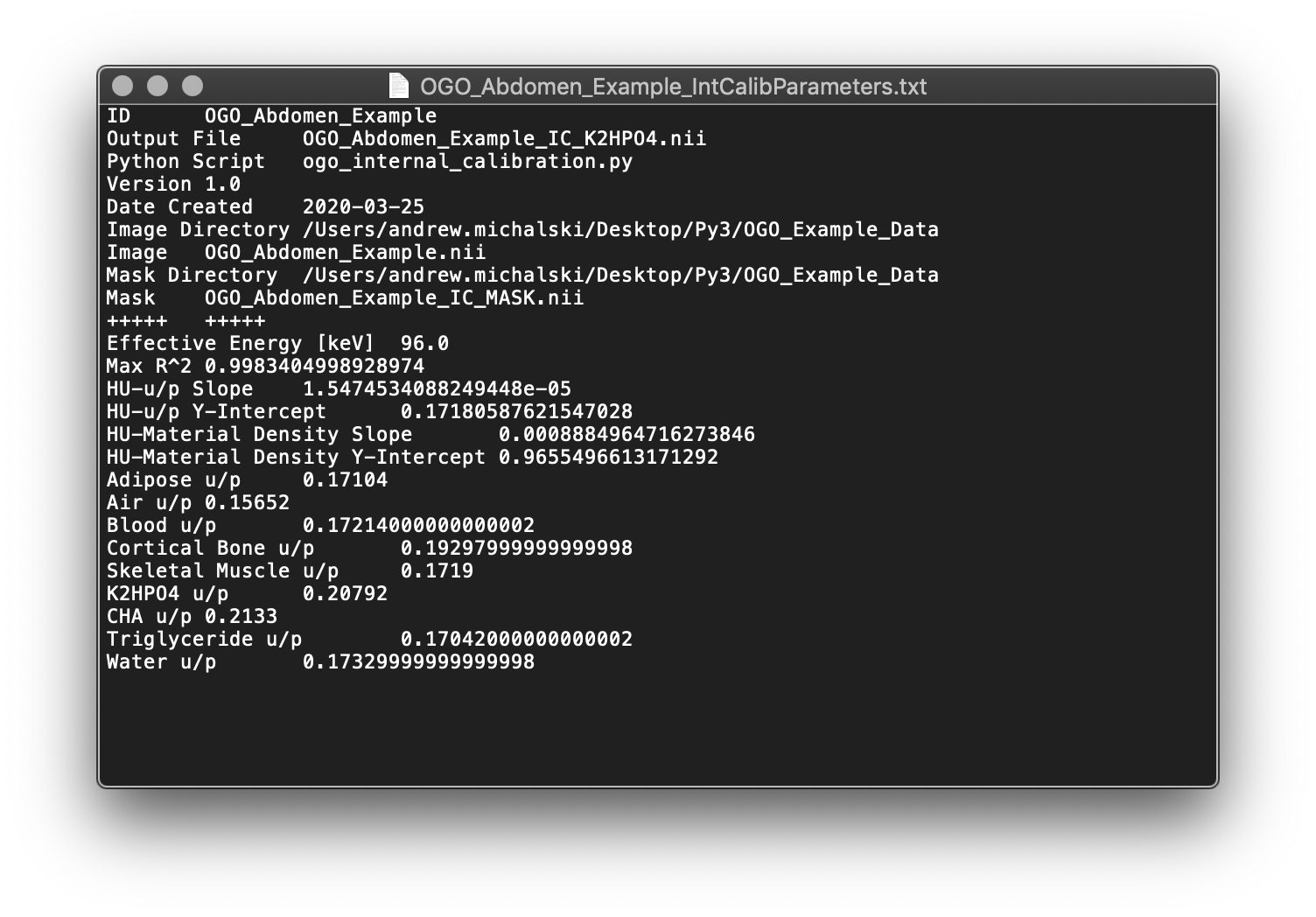
1. **_IntCalibParameters.txt* – Text file containing all of the internal density calibration parameters, as seen below.

2.**_IC_K2HPO4.nii* – K_2_HPO_4_ bone equivalent density calibrated image in NIFTI format. This file is used for BMD analysis and FE model generation.
